# 3-Chloro-6-[2-(propan-2-yl­idene)hydrazin­yl]pyridazine

**DOI:** 10.1107/S1600536810034239

**Published:** 2010-08-28

**Authors:** Abdul Qayyum Ather, M. Nawaz Tahir, Misbahul Ain Khan, Muhammad Makshoof Athar

**Affiliations:** a Department of Chemistry, Islamia University, Bahawalpur, Pakistan; b Applied Chemistry Research Center, PCSIR Laboratories Complex, Lahore 54600, Pakistan; c Department of Physics, University of Sargodha, Sargodha, Pakistan; d Institute of Chemistry, University of the Punjab, Lahore, Pakistan

## Abstract

In the title compound, C_7_H_9_ClN_4_, the 3-chloro-6-hydrazinylpyridazine unit is planar (r.m.s. deviation = 0.0219 Å) and is oriented at a dihedral angle 4.66 (27)° with respect to the propan-2-yl­idene group. In the crystal, the mol­ecules are linked into non-planar dimers due to a crystallographic twofold rotation *via* N—H⋯N hydrogen bonds with *R*_2_^2^(8) graph-set ring motifs.

## Related literature

For a related structure, see: Ather *et al.* (2010 ▶). For graph-set notation, see: Bernstein *et al.* (1995 ▶).
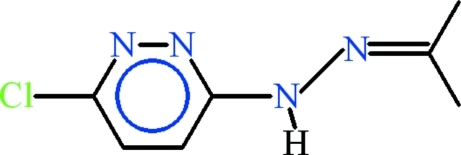


## Experimental

### 

#### Crystal data

C_7_H_9_ClN_4_
*M* *_r_* = 184.63
Monoclinic, 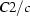

*a* = 20.6635 (19) Å
*b* = 7.8202 (6) Å
*c* = 11.3266 (8) Å
β = 94.140 (3)°
*V* = 1825.5 (3) Å^3^
*Z* = 8
Mo *K*α radiation
μ = 0.37 mm^−1^
*T* = 296 K
0.30 × 0.15 × 0.14 mm

#### Data collection

Bruker Kappa APEXII CCD diffractometer
Absorption correction: multi-scan (*SADABS*; Bruker, 2005 ▶) *T*_min_ = 0.982, *T*_max_ = 0.988
6699 measured reflections
1658 independent reflections
1176 reflections with *I* > 2σ(*I*)
*R*_int_ = 0.034

#### Refinement

*R*[*F* ^2^ > 2σ(*F* ^2^)] = 0.042
*wR*(*F* ^2^) = 0.114
*S* = 1.02
1658 reflections
111 parameters
H-atom parameters constrained
Δρ_max_ = 0.19 e Å^−3^
Δρ_min_ = −0.26 e Å^−3^

### 

Data collection: *APEX2* (Bruker, 2009 ▶); cell refinement: *SAINT* (Bruker, 2009 ▶); data reduction: *SAINT*; program(s) used to solve structure: *SHELXS97* (Sheldrick, 2008 ▶); program(s) used to refine structure: *SHELXL97* (Sheldrick, 2008 ▶); molecular graphics: *ORTEP-3 for Windows* (Farrugia, 1997 ▶) and *PLATON* (Spek, 2009 ▶); software used to prepare material for publication: *WinGX* (Farrugia, 1999 ▶) and *PLATON*.

## Supplementary Material

Crystal structure: contains datablocks global, I. DOI: 10.1107/S1600536810034239/si2291sup1.cif

Structure factors: contains datablocks I. DOI: 10.1107/S1600536810034239/si2291Isup2.hkl

Additional supplementary materials:  crystallographic information; 3D view; checkCIF report

## Figures and Tables

**Table 1:** Hydrogen-bond geometry (Å, °)

*D*—H⋯*A*	*D*—H	H⋯*A*	*D*⋯*A*	*D*—H⋯*A*
N3—H3*A*⋯N1^i^	0.86	2.33	3.083 (2)	146

Symmetry code: (i) 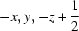.

**Fig. 1.**
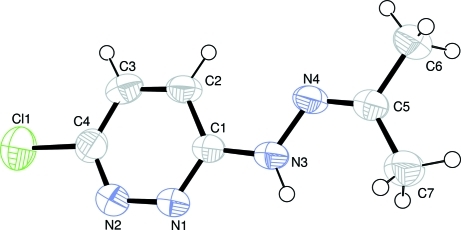
View of the title compound with the atom numbering scheme. The thermal ellipsoids are drawn at the 50% probability level. H-atoms are shown as small spheres of arbitrary radii.

**Fig. 2.**
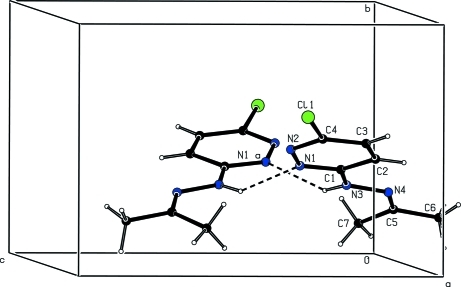
Perspective view of a pair of symmetry related title molecules with the N—H···N hydrogen bonds indicated by dashed lines [Symmetry code: a = - x, y, - z + 1/2].

## supplementary crystallographic information

### Comment

In continuation to 3-chloro-6-hydrazinylpyridazine derivatives (Ather *et al.*, 2010), the title compound (I, Fig. 1) is being reported here.

In (I), the 3-chloro-6-hydrazinylpyridazine moiety A (C1—C4/N1—N4/CL1) is planar with r. m. s. deviation of 0.0219 Å. The propyl group B(C5/C6/C7) is certainly planar. The dihedral angle between A/B is 4.66 (27)°. The title compound consists of non-planar dimers due to N—H···N type of H-bonding (Table 1, Fig. 2) with *R*_2_^2^(8) ring motif (Bernstein *et al.*, 1995). The dimers are formed due to a crystallographic twofold rotation axis parallel *b* and located in *c* = 1/4.

### Experimental

3-Chloro-6-hydrazinylpyridazine (0.5 g, 3.46 mmol), dissolved in acetone was refluxed for 15 min. The unreacted acetone was distilled off yielding in crude material. The product was re-crystallized in alcohol to affoard the colorless needles of (I).

### Refinement

The H-atoms were positioned geometrically (N–H = 0.86, C–H = 0.93–0.96 Å) and were included in the refinement in the riding model approximation, with *U*_iso_(H) = x*U*_eq_(C, N), where x = 1.5 for methyl and x = 1.2 for all other H-atoms.

### Crystal data

**Table:**

C_7_H_9_ClN_4_	*F*(000) = 768
*M**_r_* = 184.63	*D*_x_ = 1.344 Mg m^−^^3^
Monoclinic, *C*2/*c*	Mo *K*α radiation, λ = 0.71073 Å
Hall symbol: -C 2yc	Cell parameters from 1176 reflections
*a* = 20.6635 (19) Å	θ = 2.0–25.3°
*b* = 7.8202 (6) Å	µ = 0.37 mm^−^^1^
*c* = 11.3266 (8) Å	*T* = 296 K
β = 94.140 (3)°	Needle, colorless
*V* = 1825.5 (3) Å^3^	0.30 × 0.15 × 0.14 mm
*Z* = 8	

### Data collection

**Table:**

Bruker Kappa APEXII CCD diffractometer	1658 independent reflections
Radiation source: fine-focus sealed tube	1176 reflections with *I* > 2σ(*I*)
graphite	*R*_int_ = 0.034
Detector resolution: 8.10 pixels mm^-1^	θ_max_ = 25.3°, θ_min_ = 2.0°
ω scans	*h* = −23→24
Absorption correction: multi-scan (*SADABS*; Bruker, 2005)	*k* = −9→8
*T*_min_ = 0.982, *T*_max_ = 0.988	*l* = −11→13
6699 measured reflections	

### Refinement

**Table:**

Refinement on *F*^2^	Primary atom site location: structure-invariant direct methods
Least-squares matrix: full	Secondary atom site location: difference Fourier map
*R*[*F*^2^ > 2σ(*F*^2^)] = 0.042	Hydrogen site location: inferred from neighbouring sites
*wR*(*F*^2^) = 0.114	H-atom parameters constrained
*S* = 1.02	*w* = 1/[σ^2^(*F*_o_^2^) + (0.0466*P*)^2^ + 1.083*P*] where *P* = (*F*_o_^2^ + 2*F*_c_^2^)/3
1658 reflections	(Δ/σ)_max_ < 0.001
111 parameters	Δρ_max_ = 0.19 e Å^−^^3^
0 restraints	Δρ_min_ = −0.26 e Å^−^^3^

### Special details

**Table:**

Geometry. Bond distances, angles *etc*. have been calculated using the rounded fractional coordinates. All su's are estimated from the variances of the (full) variance-covariance matrix. The cell e.s.d.'s are taken into account in the estimation of distances, angles and torsion angles
Refinement. Refinement of *F*^2^ against ALL reflections. The weighted *R*-factor *wR* and goodness of fit *S* are based on *F*^2^, conventional *R*-factors *R* are based on *F*, with *F* set to zero for negative *F*^2^. The threshold expression of *F*^2^ > σ(*F*^2^) is used only for calculating *R*-factors(gt) *etc*. and is not relevant to the choice of reflections for refinement. *R*-factors based on *F*^2^ are statistically about twice as large as those based on *F*, and *R*- factors based on ALL data will be even larger.

### Fractional atomic coordinates and isotropic or equivalent isotropic displacement parameters (Å^2^)

**Table:**

	*x*	*y*	*z*	*U*_iso_*/*U*_eq_	
Cl1	0.24961 (3)	0.56355 (11)	0.22863 (7)	0.0864 (3)	
N1	0.08033 (8)	0.3556 (2)	0.21840 (14)	0.0503 (6)	
N2	0.13843 (9)	0.4249 (2)	0.25439 (15)	0.0547 (7)	
N3	0.00430 (8)	0.2720 (2)	0.07587 (14)	0.0528 (6)	
N4	−0.01436 (9)	0.2424 (2)	−0.04230 (13)	0.0497 (6)	
C1	0.06381 (10)	0.3389 (3)	0.10295 (16)	0.0429 (7)	
C2	0.10598 (11)	0.3850 (3)	0.01608 (18)	0.0519 (8)	
C3	0.16374 (12)	0.4516 (3)	0.0531 (2)	0.0583 (9)	
C4	0.17733 (11)	0.4706 (3)	0.1745 (2)	0.0537 (8)	
C5	−0.06866 (11)	0.1693 (3)	−0.06689 (17)	0.0469 (7)	
C6	−0.08681 (12)	0.1354 (3)	−0.19521 (18)	0.0628 (9)	
C7	−0.11442 (12)	0.1098 (3)	0.0200 (2)	0.0663 (9)	
H2	0.09422	0.36991	−0.06405	0.0622*	
H3	0.19359	0.48381	−0.00039	0.0699*	
H3A	−0.02122	0.24848	0.13034	0.0634*	
H6A	−0.05590	0.18936	−0.24248	0.0942*	
H6B	−0.12926	0.18084	−0.21605	0.0942*	
H6C	−0.08688	0.01439	−0.20933	0.0942*	
H7A	−0.09286	0.02922	0.07329	0.0994*	
H7B	−0.15116	0.05591	−0.02135	0.0994*	
H7C	−0.12876	0.20582	0.06396	0.0994*	

### Atomic displacement parameters (Å^2^)

**Table:**

	*U*^11^	*U*^22^	*U*^33^	*U*^12^	*U*^13^	*U*^23^
Cl1	0.0588 (5)	0.1068 (6)	0.0929 (6)	−0.0195 (4)	0.0002 (3)	−0.0051 (4)
N1	0.0506 (11)	0.0648 (12)	0.0357 (10)	−0.0027 (9)	0.0055 (8)	0.0008 (8)
N2	0.0537 (12)	0.0650 (12)	0.0451 (10)	−0.0025 (9)	0.0026 (9)	−0.0020 (9)
N3	0.0547 (12)	0.0714 (12)	0.0332 (9)	−0.0086 (10)	0.0089 (8)	−0.0016 (9)
N4	0.0558 (12)	0.0615 (11)	0.0321 (9)	0.0040 (9)	0.0046 (8)	−0.0021 (8)
C1	0.0483 (13)	0.0454 (11)	0.0355 (11)	0.0028 (9)	0.0062 (9)	−0.0001 (9)
C2	0.0598 (15)	0.0606 (14)	0.0363 (11)	−0.0014 (11)	0.0109 (10)	0.0009 (10)
C3	0.0591 (16)	0.0653 (15)	0.0527 (14)	−0.0003 (12)	0.0197 (11)	0.0042 (12)
C4	0.0485 (14)	0.0565 (13)	0.0562 (14)	0.0010 (10)	0.0056 (10)	0.0012 (11)
C5	0.0535 (14)	0.0484 (12)	0.0389 (11)	0.0056 (10)	0.0040 (10)	−0.0003 (10)
C6	0.0659 (16)	0.0787 (17)	0.0431 (12)	0.0017 (13)	−0.0011 (11)	−0.0038 (12)
C7	0.0731 (17)	0.0757 (16)	0.0507 (14)	−0.0198 (14)	0.0088 (12)	−0.0024 (12)

### Geometric parameters (Å, °)

**Table:**

Cl1—C4	1.733 (2)	C5—C6	1.498 (3)
N1—N2	1.353 (2)	C5—C7	1.488 (3)
N1—C1	1.334 (2)	C2—H2	0.9300
N2—C4	1.303 (3)	C3—H3	0.9300
N3—N4	1.385 (2)	C6—H6A	0.9600
N3—C1	1.351 (3)	C6—H6B	0.9600
N4—C5	1.272 (3)	C6—H6C	0.9600
N3—H3A	0.8600	C7—H7A	0.9600
C1—C2	1.408 (3)	C7—H7B	0.9600
C2—C3	1.341 (3)	C7—H7C	0.9600
C3—C4	1.391 (3)		
			
N1···N3^i^	3.083 (2)	H3A···C7	2.4700
N2···C2^ii^	3.425 (3)	H3A···H7A	2.3300
N3···N1^i^	3.083 (2)	H3A···H7C	2.3200
N1···H3A^i^	2.3300	H3A···N1^i^	2.3300
N1···H6C^iii^	2.9000	H6A···H6A^vii^	2.3300
N1···H7C^i^	2.8500	H6B···H7B	2.4800
N2···H7C^i^	2.7000	H6B···C4^v^	2.9500
N2···H2^ii^	2.8100	H6C···N1^iii^	2.9000
N3···H7A	2.7600	H6C···C1^iii^	3.0400
N3···H7C	2.7900	H6C···H7A^vi^	2.4800
N4···H2	2.4800	H7A···N3	2.7600
C2···N2^iv^	3.425 (3)	H7A···H3A	2.3300
C3···C5^v^	3.566 (3)	H7A···C6^viii^	2.9200
C5···C3^v^	3.566 (3)	H7A···H6C^viii^	2.4800
C1···H6C^iii^	3.0400	H7B···H6B	2.4800
C3···H7C^v^	3.0500	H7C···N3	2.7900
C4···H6B^v^	2.9500	H7C···H3A	2.3200
C6···H7A^vi^	2.9200	H7C···N1^i^	2.8500
C7···H3A	2.4700	H7C···N2^i^	2.7000
H2···N4	2.4800	H7C···C3^v^	3.0500
H2···N2^iv^	2.8100		
			
N2—N1—C1	119.56 (17)	C1—C2—H2	121.00
N1—N2—C4	118.63 (17)	C3—C2—H2	121.00
N4—N3—C1	117.99 (16)	C2—C3—H3	121.00
N3—N4—C5	117.79 (16)	C4—C3—H3	121.00
N4—N3—H3A	121.00	C5—C6—H6A	109.00
C1—N3—H3A	121.00	C5—C6—H6B	109.00
N1—C1—N3	115.14 (17)	C5—C6—H6C	109.00
N1—C1—C2	122.19 (19)	H6A—C6—H6B	109.00
N3—C1—C2	122.66 (17)	H6A—C6—H6C	109.00
C1—C2—C3	117.56 (19)	H6B—C6—H6C	109.00
C2—C3—C4	117.5 (2)	C5—C7—H7A	109.00
Cl1—C4—C3	120.12 (18)	C5—C7—H7B	109.00
N2—C4—C3	124.5 (2)	C5—C7—H7C	109.00
Cl1—C4—N2	115.37 (17)	H7A—C7—H7B	109.00
C6—C5—C7	117.4 (2)	H7A—C7—H7C	110.00
N4—C5—C6	116.55 (19)	H7B—C7—H7C	109.00
N4—C5—C7	126.04 (18)		
			
C1—N1—N2—C4	−1.3 (3)	N3—N4—C5—C6	−178.64 (18)
N2—N1—C1—N3	−178.55 (17)	N3—N4—C5—C7	−0.8 (3)
N2—N1—C1—C2	2.5 (3)	N1—C1—C2—C3	−1.7 (3)
N1—N2—C4—Cl1	178.26 (14)	N3—C1—C2—C3	179.5 (2)
N1—N2—C4—C3	−0.7 (3)	C1—C2—C3—C4	−0.3 (3)
C1—N3—N4—C5	175.6 (2)	C2—C3—C4—Cl1	−177.43 (19)
N4—N3—C1—N1	−176.46 (17)	C2—C3—C4—N2	1.5 (4)
N4—N3—C1—C2	2.5 (3)		

Symmetry codes: (i) −*x*, *y*, −*z*+1/2; (ii) *x*, −*y*+1, *z*+1/2; (iii) −*x*, −*y*, −*z*; (iv) *x*, −*y*+1, *z*−1/2; (v) −*x*, −*y*+1, −*z*; (vi) *x*, −*y*, *z*−1/2; (vii) −*x*, *y*, −*z*−1/2; (viii) *x*, −*y*, *z*+1/2.

### Hydrogen-bond geometry (Å, °)

**Table:**

*D*—H···*A*	*D*—H	H···*A*	*D*···*A*	*D*—H···*A*
N3—H3A···N1^i^	0.86	2.33	3.083 (2)	146

Symmetry codes: (i) −*x*, *y*, −*z*+1/2.
